# Blood biomarkers of disease activity in pediatric idiopathic nephrotic syndrome: A prospective study 

**DOI:** 10.5414/CNP104S13

**Published:** 2025-11-28

**Authors:** Matjaž Kopač, Aleš Jerin, Tanja Kersnik Levart, Joško Osredkar

**Affiliations:** 1 Department of Nephrology, Division of Pediatrics,; 2 Institute of Clinical Chemistry and Biochemistry, University Medical Center Ljubljana, and; 3 Faculty of Pharmacy, University of Ljubljana, Ljubljana, Slovenija

**Keywords:** anti-nephrin antibodies, vitamins, eosinophilic cationic protein, nephrotic syndrome, children

## Abstract

Introduction: The etiology and pathogenesis of idiopathic nephrotic syndrome (INS) in children remains incompletely understood. We investigated correlations of blood concentrations of eosinophilic cationic protein (ECP), some vitamins, and anti-nephrin antibodies with disease activity of INS in Slovenian children. Materials and methods: In this prospective, single-center study, we took sequential blood samples from children with INS at disease onset or relapse (before corticosteroid (CS) treatment), at time of remission, and after discontinuation of CS treatment, whenever feasible. We performed the quantitative detection of anti-nephrin antibodies in patients serum with enzyme-linked immuno-sorbent assay, blood concentration measurements of ECP and vitamins with standard laboratory methods and statistical analysis with ANOVA. Results: We included 17 children with INS (15 boys and 2 girls). We detected statistically significantly highest ECP concentrations at disease onset or relapse, lowest vitamin E concentrations in remission after CS treatment and highest vitamin A concentrations at time of remission achievement. We also detected decreased levels of vitamin D at times of disease onset, relapse and remission achievement. However, we did not detect anti-nephrin antibodies in any serum sample. Conclusion: We confirmed significant concentration variations of ECP and vitamins E, A, and D at different stages of INS disease activity. These findings suggest their potential role in the etiology of INS and make these molecules as candidates for biomarkers of disease activity. We did not confirm the pathogenic role of anti-nephrin antibodies in our pediatric population.

## Introduction 

Idiopathic nephrotic syndrome (INS) in children presents with significant morbidity and has a variable disease course. This article aims to elucidate the influence of various potential biomarkers at different disease stages, the roles of vitamins, eosinophilic cationic protein (ECP) and anti-nephrin antibodies in INS. 

Despite extensive research, the etiology and pathogenesis of INS remains incompletely understood. Genetic predispositions and environmental triggers are considered contributing factors. Mutations in genes such as *NPHS1* (encoding nephrin), *NPHS2* (podocin), and in many others have been implicated in genetic cases of nephrotic syndrome. Apart from these cases with a single-gene defect, most cases are probably immune-mediated and caused by the presence of a still unidentified circulating factor [1]. 

The clinical manifestation of INS consists of edema, hypoalbuminemia, and nephrotic range proteinuria which have several consequences due to hemodynamic effects and loss of essential circulating proteins. These patients are usually treated with immunosuppressive drugs without knowing the exact mechanism and are classified according to response to treatment with corticosteroids (CS). The research efforts are directed towards understanding of the underlying injury at a molecular level that could enable treatment adaptations according to the likely pathogenetic mechanism. Current classification of INS is based on observational features, mainly according to response to CS or according to microscopy findings on renal biopsy. The most common among these, especially in children, is minimal change disease (MCD), revealing effacement of podocyte foot processes on electron microscopy. The second most common biopsy finding is focal segmental glomerulosclerosis (FSGS), characterized by the presence of focal, segmental, chronic, and sclerotic injury in the glomerulus [1]. 

According to current knowledge, INS is primarily a disorder of the glomerular filtration barrier, leading to massive proteinuria. The role of immune dysregulation is significant, with alterations in T-cell and B-cell function contributing to disease manifestation. Podocyte injury and the loss of glomerular basement membrane integrity are central to pathogenesis [2]. 

At disease onset, elevated levels of proteinuria and hypoalbuminemia are primary indicators. Biomarkers such as serum creatinine, estimated glomerular filtration rate (eGFR), and urinary protein-to-creatinine ratio are crucial for diagnosis and assessing disease severity. Relapse is marked by the reappearance of proteinuria. Monitoring serum albumin, urinary protein levels and eGFR helps in assessing relapse severity [3]. Additionally, increased levels of proinflammatory cytokines such as interleukin-6 (IL-6) and tumor necrosis factor-alpha (TNF-α) have been observed [4]. Remission is characterized by the normalization of proteinuria and serum albumin levels. Biomarkers such as anti-nephrin antibodies may decrease, and there is a reduction in proinflammatory cytokines [5]. After discontinuation of CS treatment, monitoring for relapse is critical. Persistent alterations in urinary protein levels, serum creatinine, and cytokine profiles can indicate ongoing subclinical disease activity [6]. 

## Materials and methods 

In this prospective single-center study with 18 months duration, we took sequential blood samples from children with INS at the following time points (whenever feasible):

- at disease onset or relapse (before initiation of CS treatment),
- at the time of remission achievement with CS treatment,
- at the time of remission, persisting after discontinuation of CS treatment, at least 2 months after remission achievement.

Written informed consent was obtained prior to sample collections from patients’ parents and patients if they were over 14 years of age. The study was approved by the Slovenian National Committee for Medical Ethics (decision number 0120-501/2022/3). 

We performed the quantitative detection of anti-nephrin (anti-NPHS1) antibodies in patients’ serum with a kit based on enzyme-linked immuno-sorbent assay (ELISA) technology. Anti-NPHS1 ELISA Kit (ABX572112, Abbexa Ltd, Cambridge, UK) operates on the sandwich ELISA principle. In this method, a capture antibody specific to the target antigen is pre-coated onto a microplate. When a sample is added, any antigen present binds to this capture antibody. After washing away unbound substances, a detection antibody – also specific to the antigen and linked to an enzyme – is introduced, forming a “sandwich” with the antigen between the two antibodies. Following another wash step to remove unbound detection antibody, a substrate is added. The enzyme catalyzes a reaction with the substrate, producing a measurable signal, typically a color change, which is proportional to the amount of antigen in the sample. Analytical sensitivity of the test is 1.22 µg/L. 

Vitamins A (retinol), D (25-hydroxyvitamin D), and E (tocopherols) were measured using a competitive chemiluminescent immunoassay on the Abbott Architect automated analyzer (Architect analyzer, Abbott Diagnostics, Lake Forest, IL, USA). This method is based on a competitive immunoassay principle where the vitamins in the patient sample compete with a chemiluminescent-labeled analog for binding to a specific antibody. The labeled analog and the specific antibody are pre-incubated in the reaction well. When the patient sample is added, the vitamin present competes with the labeled analog for the limited antibody binding sites. The amount of labeled analog bound is inversely proportional to the concentration of vitamin in the sample. The limit of quantitation for 25-hydroxyvitamin D is 10 nmol/L. Blood concentration measurements of ECP were measured in a routine manner using automated machine method. 

Statistical analysis was done with the ANOVA method. Statistical significance of concentration differences of studied molecules in plasma samples was presented as p-value, with value less than 0.05 considered statistically significant. 

## Results 

**Figure 1:**
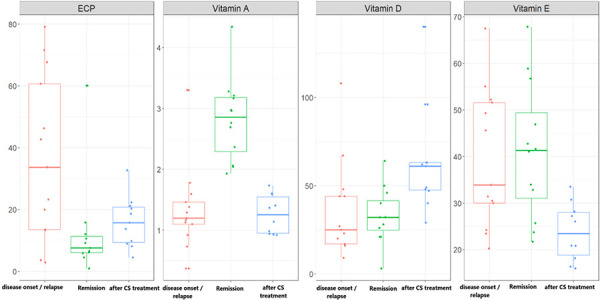
Blood concentrations of eosinophilic cationic protein and studied vitamins in various phases of idiopathic nephrotic syndrome disease activity. Results are presented with the use of boxplots where middle lines present mean values, lower end of boxes present first quartiles (25%), and upper end of boxes present third quartiles (75%). Dots present individual measurements. ECP = eosinophilic cationic protein; CS = corticosteroids. Results of measurements (on Y-axis) are presented in following units, including reference ranges for all age groups (in brackets): ECP (3.6 – 17.3 µg/L); vitamin A (0.7 – 1.4 µmol/L); vitamin D (32 – 165 nmol/L); vitamin E (5- 20 µmol/L).

17 patients with INS, aged between 3 and 18 years, were included in the study, 15 boys and 2 girls. Twelve of them had a kidney biopsy and all of them had MCD, with no other abnormalities suggesting autoimmune etiology of the disease. Four of them were new cases at disease onset, 10 had relapse and 3 of them were in stable remission. Two of the included children were receiving CS treatment, 3 of them vitamin D and 7 of them other immunosuppressive drugs (4 of them mycophenolate-mofetil (MMF), 1 cyclosporine, and 2 tacrolimus) at relapse. The correlations of ECP and studied vitamins with disease activity are presented in Table 1 and schematically in Figure 1. 

We did not detect anti-nephrin antibodies within limits of detection with commercially available laboratory kit we used, which was based on ELISA technology, in any serum sample analyzed. 

## Discussion 

### Role of vitamins and ECP 

We detected statistically significantly increased vitamin A concentrations at time of remission, compared to disease onset or relapse and time after CS treatment discontinuation, suggesting its role in immune function and maintaining the structural integrity of the glomerular filtration barrier. Serum vitamin A and vitamin A protein carriers, retinyl esters, and vitamin E concentrations were increased in a study on 33 patients with chronic glomerulonephritis and nephrotic syndrome with preserved kidney function that were most likely a consequence of increased protein and lipid synthesis in the liver due to albumin loss in urine. In addition, indirect relationships were detected between serum vitamin E and albumin concentrations. These results showed the presence of hypervitaminosis of vitamins A and E in patients with nephrotic syndrome [7]. However, we detected increased vitamin A concentrations at time of remission, suggesting either a time delay of vitamin A peak concentrations or another, yet unknown, influence of this vitamin on disease activity. It is worth mentioning a published case report of a 3-year-old child with acute promyelocytic leukemia who developed transient nephrotic-range proteinuria during all-trans retinoic acid treatment (a vitamin A derivative), complicated by retinoic acid syndrome, characterized by an inflammatory reaction with capillary leakage and myeloid cell tissue invasion causing cardiopulmonary symptoms and, sometimes, acute kidney injury (AKI). All-trans retinoic acid was temporarily discontinued, and the patient was treated with dexamethason, followed by complete resolution of proteinuria and preservation of kidney function [8]. 

We detected decreased levels of vitamin D at disease onset or relapse that may suggest the role of relative vitamin D deficiency in increasing disease susceptibility. Vitamin D has been reported as crucial for immune regulation and maintaining renal health. Deficiency in vitamin D, a common comorbidity in children with INS, has been associated with increased disease activity and relapse rates in INS [9], similar to results of our study. This was supported by another cross-sectional study in 96 children with INS aged 1 – 18 years revealing that 77.1% of these patients had vitamin D deficiency. 75% of children with the first episode of INS, 80% of those with infrequently relapsing INS and 91% of those with frequently relapsing nephrotic syndrome had vitamin D deficiency. A negative correlation between vitamin D concentration and duration of illness was found [10]. In addition, vitamin D and vitamin D receptor (VDR) deficiency exacerbates several other experimental autoimmune diseases such as inflammatory bowel disease, a consequence of an immune-mediated attack by pathogenic T cells that overproduce IL-17 and IFN-gamma and a few regulatory cells. In the absence of vitamin D and the VDR, autoimmunity occurs in the gastrointestinal tract due to increased numbers of IL-17 and IFN-γ-secreting T cells and a concomitant reduction in regulatory T cells [11]. 

We confirmed statistically significantly increased vitamin E concentrations at disease onset or relapse and at time of remission, compared to remission after discontinuation of CS treatment, suggesting its activation in order to mitigate oxidative stress, which is obviously elevated in this disease. Vitamin E, with its antioxidant properties, may help mitigate oxidative stress associated with INS. Its role in reducing lipid peroxidation and stabilizing cellular membranes is beneficial in disease management. A recent meta-analysis, performed to investigate the alteration of serum levels of malondialdehyde (MDA), vitamin C and E in INS revealed that active steroid-sensitive nephrotic syndrome patients had similar concentrations of serum vitamin C and E as controls. However, INS subjects in the remission stage demonstrated significantly higher concentrations of serum MDA, lower concentrations of serum vitamin C and similar concentrations of serum vitamin E compared to controls. Therefore, it might well be that the serum concentrations of vitamin C and E are associated with the responsiveness of INS to CS [12]. 

Regarding studying these associations, we are aware of the challenges of measuring serum concentrations of vitamins A, D, and E due to their binding to proteins that are lost with urine in INS. In addition, their serum concentrations are subjected to variability, often independently of their actual tissue amounts. However, all of the included children had nephrotic range proteinuria as well as some degree of hypoalbuminemia at disease onset or relapse (before CS treatment) and absence of proteinuria at remission, according to INS definitions [3]. For this reason we decided not to analyze degree of proteinuria and/or hypoalbuminemia. In addition, we detected increased concentrations of ECP, vitamin E, and vitamin A in the initial phase of INS (disease onset or relapse and remission) despite often concomitant hypoalbuminemia, but not in the inactive phase, in long-term remission after CS treatment (representing a sort of control group), with normal serum albumin levels. However, due to the possibility that vitamin concentrations reflect their physiologic changes in transport and distribution and not just disease activity, we are aware of limitations of these results. 

We detected also a statistically significantly increased ECP concentration at disease onset or relapse compared to remission. This has not been described before and may suggest its pathogenic role upon podocyte slit diaphragm as well as eosinophilic activation during disease. 

The mechanism by which a circulating factor, which has not been yet identified, causes an injury to the glomerular capillary wall in MCD, is incompletely understood. The glomerular capillary wall separates the capillary lumen from Bowman’s space and consists of the fenestrated endothelium, the glomerular basement membrane (GBM), and the epithelium with a slit diaphragm between podocyte foot processes. The endothelium and the GBM are strongly anionic with the electronegative charges caused by sialic acid and heparan sulfate. In patients with MCD, the glomerular permeability factor could abolish the anionic properties of the GBM [13]. We investigated whether ECP, with its cationic charge, could be a molecule responsible for disruption of the GBM anionic charge. According to our findings, this may be the case, however, it could be an epiphenomenon and not necessarily a cause of the disease. 

### Role of anti-nephrin antibodies 

Anti-nephrin antibodies target nephrin, a critical component of the slit diaphragm in podocytes. The presence of these antibodies in INS suggests an autoimmune component contributing to podocyte injury and proteinuria. Understanding the role of these antibodies can help in developing targeted therapies [14]. 

Antibodies targeting nephrin have been shown to cause nephrotic range proteinuria when administered in animal models [15, 16]. In a study of 62 adults and children with MCD without a genetic cause, circulating anti-nephrin antibodies were identified in 29% of patients with active disease. Antibody titers were decreased or absent during clinical response to treatment. One female patient with childhood-onset MCD with steroid-dependent nephrotic syndrome progressed to FSGS and end-stage kidney disease. After renal transplantation, she quickly developed recurrent nephrotic range proteinuria that rapidly responded to treatment with plasmapheresis and rituximab. Serum samples prior to transplantation and plasmapheresis initiation tested positive by ELISA for anti-nephrin antibodies. However, serum samples after successful treatment (one month and one year post-transpant) were negative for anti-nephrin antibodies [17]. 

In another multicenter study of 539 patients (357 adults and 182 children) with biopsy-proven glomerular disease, circulating anti-nephrin antibodies were detected in 44% of adults with MCD, 9% of those with primary FSGS, and rarely in those with other diseases. Among the patients with MCD, the prevalence of anti-nephrin antibodies was 61% in those with nephrotic range proteinuria and 69% in those without immunosuppressive therapy. Autoantibody levels correlated with disease activity. In 2 patients with anti-nephrin-associated podocytopathy, treatment with rituximab led to clinical remission and disappearance of anti-nephrin autoantibodies [18]. These findings suggest that an autoimmune etiology may be responsible in a subset of patients with MCD [13]. 

However, we did not detect anti-nephrin antibodies within limits of detection with commercially available laboratory kit we used, which was based on ELISA technology, in any serum sample analyzed. This result implies that, at least within the detectable boundaries of the ELISA technique employed, autoantibodies against nephrin may not be a major contributor to the pathophysiology of the disease in our pediatric sample. On the other hand, we are aware that some other studies used more sophisticated, validated, and specially designed autoantibodies against nephrin resulting in positive results. This may be considered as a limitation of our study. 

The complexity of INS, especially in cases that are resistant to CS, has been brought to light by recent studies. According to such a study, 35.5% of steroid-resistant INS patients had anti-nephrin antibodies, which are linked to worsening early symptoms but improved immunosuppressive response [19]. This gives support to the idea that INS has a subset of autoimmune podocytopathy. With possible contributions from innate immunity, the immune system – in particular T cells – seems to be essential throughout the disease’s active phase [20]. These results point to a diverse illness landscape that calls for individualized diagnostic and therapeutic strategies based on clinical, immunological, and genetic variables. The pathophysiology of INS may be more heavily influenced by other immunological pathways, such as cytokine imbalances, podocyte damage caused by circulating substances (including suPAR), or T-cell dysfunction. Since there are no detectable anti-nephrin antibodies, podocyte destruction in our patient group is most likely caused by other processes. 

## Conclusion 

INS in children involves complex interactions between immunological and environmental factors. Vitamins A, D, and E have immunomodulatory and antioxidant effects that may influence disease outcomes. Understanding the multifaceted pathogenesis and monitoring biomarkers can enhance therapeutic strategies and improve prognosis in children with INS. Although we did not find anti-nephrin antibodies in any of the serum samples, the sample size may have an impact on significance of this finding. To definitively demonstrate their absence, larger research or different approaches may be required. 

## Acknowledgment 

The authors wish to thank the doctors who agreed to include 1 or 2 of their patients and nurses for taking blood samples and sending them to laboratory, all from the Department of Nephrology, Division of Pediatrics. Authors also wish to thank the employees of the Center for Clinical Research and of the Institute of Clinical Chemistry and Biochemistry, all from University Medical Centre Ljubljana, for their contributions. 

## Authors’ contributions 

Conceptualization: M.K.; methodology: M.K.. and J.O; Validation: J.O. and A.J.; Formal analysis: M.K., T.K.L. and J.O.; Investigation: A.J. and M.K.; Resources: M.K. and J.O.; Writing – original draft preparation: M.K.; Writing – review and editing: M.K, J.O., A.J. and T.K.L.; Visualization: M.K., A.J., T.K.L. and J.O.; Supervision: M.K., T.K.L. and J.O.; Project administration: M.K. and J.O.; Funding acquisition: M.K. All authors have read and agreed to the published version of the manuscript. 

## Funding 

This research was funded by University Medical Center Ljubljana, tertiary science project, funding number 20220062. 

## Conflict of interest 

The authors declare no conflict of interest. 

**Table 1.** Correlation of blood concentrations of eosinophilic cationic protein and studied vitamins (with reference values for all age groups in brackets) with disease activity of idiopathic nephrotic syndrome. Results are presented as average (standard deviation).

	N	Disease onset/relapse	Remission	After CS treatment	Total	p
ECP (3.6 – 17.3 µg/L)	35	36.8 (26.6)*	12.7 (16.2)	16.0 (8.1)	22.7 (21.7)	0.008
Vitamin A (0.7 – 1.4 µmol/L)	35	1.3 (0.7)	2.8 (0.7)*	1.3 (0.3)	1.8 (0.9)	< 0.001
Vitamin E (5- 20 µmol/L)	35	39.6 (14.7)	41.1 (14.6)	23.8 (6.2)*	35.6 (14.6)	0.006
Vitamin D (32 – 165 nmol/L)	36	35.7 (27.3)	32.9 (15.8)	63.5 (30.6)*	43.2 (28.1)	0.012

*Statistically significant difference. N = number of samples; Total = all samples combined; ECP = eosinophilic cationic protein; CS = corticosteroids.
